# *Aedes aegypti* SGS1 is critical for *Plasmodium gallinaceum* infection of both the mosquito midgut and salivary glands

**DOI:** 10.1186/s12936-020-03537-6

**Published:** 2021-01-06

**Authors:** Bianca B. Kojin, Ines Martin-Martin, Helena R. C. Araújo, Brian Bonilla, Alvaro Molina-Cruz, Eric Calvo, Margareth L. Capurro, Zach N. Adelman

**Affiliations:** 1 Department of Entomology and Agrilife Research, Texas A&M University, College Station, TX USA; 2 Laboratory of Malaria and Vector Research, National Institute of Allergy and Infectious Diseases, National Institutes of Health, Rockville, MD 20852 USA; 3 Departamento de Parasitologia, Laboratório de Mosquitos Geneticamente Modificados, Instituto de Ciências Biomédicas, Universidade de São Paulo, São Paulo, SP 05508-000 Brazil

**Keywords:** *Aedes aegypti*, *Plasmodium gallinaceum*, Malaria, Salivary gland, SGS1, Sporozoites, Oocyst, Midguts

## Abstract

**Background:**

The invasion of the mosquito salivary glands by *Plasmodium* sporozoites is a critical step that defines the success of malaria transmission and a detailed understanding of the molecules responsible for salivary gland invasion could be leveraged towards control of vector-borne pathogens. Antibodies directed against the mosquito salivary gland protein SGS1 have been shown to reduce *Plasmodium gallinaceum* sporozoite invasion of *Aedes aegypti* salivary glands, but the specific role of this protein in sporozoite invasion and in other stages of the *Plasmodium* life cycle remains unknown.

**Methods:**

RNA interference and CRISPR/Cas9 were used to evaluate the role of *A. aegypti* SGS1 in the *P. gallinaceum* life cycle.

**Results:**

Knockdown and knockout of SGS1 disrupted sporozoite invasion of the salivary gland. Interestingly, mosquitoes lacking SGS1 also displayed fewer oocysts. Proteomic analyses confirmed the abolishment of SGS1 in the salivary gland of SGS1 knockout mosquitoes and revealed that the C-terminus of the protein is absent in the salivary gland of control mosquitoes. In silico analyses indicated that SGS1 contains two potential internal cleavage sites and thus might generate three proteins.

**Conclusion:**

SGS1 facilitates, but is not essential for, invasion of *A. aegypti* salivary glands by *P. gallinaceum* and has a dual role as a facilitator of parasite development in the mosquito midgut. SGS1 could, therefore, be part of a strategy to decrease malaria transmission by the mosquito vector, for example in a transgenic mosquito that blocks its interaction with the parasite.

## Background

Substantial progress has been made towards reducing the burden of malaria worldwide, as the global malaria mortality rate declined from 585,000 to 405,000 between 2010 and 2018 [1]. While such achievement is extraordinary, the amount of progress is slowing down. The global incidence rate (i.e. the number of cases per 1000 population) dropped 14 cases from 2010 to 2018 (71–57 cases), however since 2014 the incidence remained stationary (57 cases), highlighting that the malaria challenge remains enormous and the development of new control measures is urgent. The difficulty in vaccine development [2], the increase of mosquito resistance to insecticides [3] as well as parasite resistance to anti-malarial drugs [4] all warrant the need for additional measures of control. Malaria prevention based on vector control, more specifically on genetic control, is an interesting intervention to be explored. Therefore, a detailed understanding of mosquito-parasite interactions are of utmost importance for the development of such interventions.

Human malaria has been extensively studied in comparison to avian malaria, however the impact of the disease in birds can be devastating. A classic example is the accidental introduction of *Plasmodium relictum* to the Hawaiian Islands in the early 1800s, which is believed to be responsible for the extinction of different native forest birds [5, 6]. More recently, avian malaria has been linked to a decline in bird abundance in New Zealand [7, 8], demonstrating the importance of the disease in the avifauna. Malaria parasites in birds are widely spread, and present in almost all continents; some populations can exhibit infection rates as high as 98% within a species [9, 10]. *Plasmodium gallinaceum* is an avian malaria parasite that provides a reliable laboratory model for studying mosquito-parasite interactions and can provide valuable information applicable to *Plasmodium falciparum* [11–14].

For both avian and human malaria, the *Plasmodium* parasite life cycle inside the invertebrate host requires the ingestion of gametocytes by the female mosquito from an infected blood meal (reviewed in [15]). Soon after ingestion, gametocytes differentiate into male and female gametes and fertilize. The newly formed zygote transforms into a mobile ookinete that traverses the peritrophic matrix and the midgut epithelium to form an oocyst. Each oocyst develops thousands of sporozoites that are released into the haemolymph. These haemolymph sporozoites must invade the mosquito salivary gland to be inoculated into a human host during the next blood meal, initiating the vertebrate host cycle. Thus, the invasion of the mosquito salivary glands is crucial for transmission and provides potential new targets for the development of refractory mosquitoes for malaria control.

Evidence supports that *Plasmodium* sporozoite invasion of the salivary glands is a receptor-ligand mediated process [16, 17] and this interaction between mosquito receptor and parasite is species-specific [18]. Molecules like glycoproteins, proteoglycans and protein receptors are known to be involved in sporozoite recognition, attachment, and invasion of the salivary gland, either in a step-by-step process or in a collaborative way (reviewed in [19]). However, few molecules from mosquito salivary glands have been characterized and their roles in this crucial process are still unknown.

One candidate protein receptor for sporozoite invasion is *Aedes aegypti* salivary gland surface protein 1 (SGS1) [20]. SGS1 and other members of the SGS family are thought to have been acquired via horizontal transfer from bacterial endosymbionts [20, 21]. This appears to have occurred early in mosquito evolution, as orthologs are found in Anopheline and Aedine mosquitoes, where subsequent species-specific expansions have occurred [21]. Despite being encoded from a single exon, SGS proteins are large, typically exceeding 300 KDa, with multiple RHS-repeats (Rearrangement HotSpot) preceding a set of multipass transmembrane domains [22]. SGS proteins are known to be secreted into the saliva [22] and are a major salivary antigen [23]. SGS1 was also shown to be present on the basal plasma membrane of the medial and distal lateral lobes of the female salivary gland epithelium, regions known to be preferred for sporozoite invasion [20, 24, 25]. Inoculation of polyclonal anti-SGS1 IgG in *P. gallinaceum*-infected *A. aegypti* prior to oocyst rupture drastically inhibited sporozoite invasion of the salivary gland, indicating that SGS1 is important during this process [20]. However, subsequent reports have indicated that *A. aegypti SGS1* has several paralogs [21], two of which are also expressed in *A. aegypti* salivary glands [26]. Thus, the specific contribution of SGS1 to parasite invasion remains unknown, as well as any role SGS1 might play in other stages of the parasite invasion process.

In this work, reverse genetic approaches based on RNAi and CRISPR/Cas9 were used to elucidate the importance of SGS1 for *P. gallinaceum* infection of the mosquito midgut and invasion of the salivary gland. RNAi-based knockdown of SGS1 timed to coincide with oocyst rupture impaired sporozoite penetration in the salivary gland of *A. aegypti* mosquitoes, consistent with a potential role in this process. Surprisingly, CRISPR/Cas9-based knockout of SGS1 also reduced the number of oocysts developing on the midgut, suggesting that SGS1 has a dual role in regulating *P. gallinaceum* infection. This study is one step forward in understanding the molecules required for the successful completion of the parasite life cycle in the mosquito host and, therefore, possible targets to stop malaria transmission.

## Methods

### Mosquito rearing and infection procedures

The *A. aegypti* Higgs white-eyed strain [27] was used for RNAi knockdown experiments of SGS1 (including *P. gallinaceum* sporozoite counts in salivary glands and haemolymph, as well as mRNA analyses by RT-PCR) and were maintained in the insectary at the Institute of Biomedical Sciences, University of São Paulo, Brazil at 27 ± 2 °C, 75–80% relative humidity with a 12:12 h light:dark cycle. Larvae were fed on Tetramin® and adults had a solution of 10% sucrose ad libitum. For egg collection, pre-mated females were blood fed on anaesthetized mice. The *A. aegypti* Liverpool (LVP) strain was used for CRISPR/Cas9 knockout experiments [including *P. gallinaceum* sporozoite counts in salivary glands and oocyst counts in midgut, proteomic analyses, as well as mRNA analyses by quantitative real time PCR reaction (qRT-PCR experiments)]. Mosquitoes were kept in confined chambers at 28 °C, 60–70% humidity with a 14:10 h light:dark and had a solution of 10% sucrose ad libitum. For egg collection females were offered defibrinated sheep blood (Colorado Serum Company, Denver, CO) in an artificial feeding system and larvae were fed on Tetramin®.

### *Plasmodium gallinaceum* infection procedure

An aliquot of frozen blood containing gametocytes from *P. gallinaceum* strain 8A [28–30], was used to inoculate 7-day-old chicks (*Gallus gallus*, Granja Kunitomo, Mogi das Cruzes, Brazil) or 6-week-old White Leghorn chickens (*G. gallus*, Charles River, Norwich, USA) to establish the infection. A blood droplet was taken daily from the chicken’s feet vein, and a blood smear stained with Giemsa was used for checking parasitaemia. Chickens with parasitaemia between 3–7% were considered acceptable for proceeding to feeding. Five-to-seven-day-old mosquitoes that had been deprived of sugar for 16 h were allowed to feed to repletion on infected chickens for 15 min and only fully engorged females were used in subsequent experiments. Each infected chicken was simultaneously exposed to mosquitoes from the experimental and control groups. Fifty mosquitoes were used per group, with three biological replicates of the entire experiment. The amount of blood ingested by each group was checked in the knockout mosquitoes; 15 mosquitos were frozen individually at –20 °C until processing. Each mosquito was placed in a 1.5 mL tube with 2–4 disruption beads (Zirconia/Silica, 2.3 mm; Research Product International 9838, Mt. Prospect, USA). Following the addition of 1 mL of Drabkin reagent, tubes were homogenized for 1 min at 4000 rpm in a Bullet Blender Storm (Next Advance, Troy, USA). Homogenized samples were clarified by centrifugation for 10 min at RT (13,548 g) prior to measuring absorbance at 540 nm in a VersaMax microplate reader (Molecular Devices, San Jose, USA).

### Generation of dsRNA and gene silencing assays

A 546 bp fragment from *aaSGS1* (AAEL009993) was amplified using primers designed from the E-RNAi Webservice (www.dkfz.de/signaling/e-rnai3/idseq.php) that incorporated T7 minimum promoter sequence at their 5′ ends; to ensure specificity primers were queried against the *A. aegypti* genome on Vectorbase (www.vectorbase.org) using the blastn program. EGFP dsRNA was used as a negative control and was amplified following the same parameters. The amplification conditions were: 98 °C for 3 min followed by 30 cycles of 98 °C for 20 s, 55 °C for 30 s and 72 °C for 2 min and a final step of 10 min at 72 °C. Primers for amplifications are listed in Additional file 1: Table S1. PCR products were purified using the QIAquick PCR Purification Kit (QIAGEN, Hilden, Germany) and used as a template for dsRNA synthesis. The reaction was carried out and cleaned using the MEGAscript T7 Transcription kit (Thermo Fisher Scientific, Waltham, USA) following the manufacture’s protocol. Five-to-seven-day-old adult females were allowed to feed on *P. gallinaceum-*infected chickens. Fully engorged females were sorted and after 7 days were cold anesthetized and injected in the thorax with 3 µg of SGS1 or EGFP dsRNA diluted in 1μL water using a pulled borosilicate glass capillary needle (World Precision Instruments, Sarasota, USA) and a syringe.

### Embryonic injections and generation of knockout lines using CRISPR/Cas9

Generation of SGS1 knockout lines was performed as described [31]. Briefly, sgRNAs targeting AAEL009993 were selected using flycrispr.org (Detailed user manual is available at http://tools.flycrispr.molbio.wisc.edu/targetFinder/CRISPRTargetFinderManual.pdf) and the corresponding primers (Additional file 1: Table S1) were used in a PCR reaction with the following parameters: 98 °C for 30 s followed by 34 cycles of 98 °C for 10 s, 60 °C for 30 s and 72 °C for 15 s and a final step of 10 min at 72 °C. Amplification products were used as templates for the generation of sgRNA using Megascript T7 kit (Thermo Fisher Scientific, Waltham, USA). The efficiency of sgRNA to generate indels was tested initially by high melting resolution analyses (HRMA) performed on the DNA extracted from approximately 100 injected embryos in triplicate. For both transient assays and germline knockout experiments, injection mixes contained three sgRNAs (100 ng/μL each) and 600 ng/μL of Cas9 protein (PNA Bio, Newbury Park, USA) and were incubated at 37 °C for 20 min prior to injection. Injected embryos were either processed for DNA isolation and HRMA analysis at 24 h post injection or allowed to recover in confined chambers for 5 days and then hatched. G_0_ survivors were backcrossed to LVP and G_1_ individuals were genotyped by HRMA (Additional file 1: Table S1). PCR amplicons for individuals with a difference in melting curve were processed using the NucleoSpin Gel and PCR Clean-up kit (Macherey–Nagel, Duren, Germany) and Sanger sequenced at the Laboratory for Genomic Technologies (Institute for Plant Genomics and Biotechnology, Texas A&M University). Following identification of out-of-frame mutations in SGS1, individuals of the same genotype were pooled and backcrossed into the LVP strain for 5 consecutive generations in order to both reduce the prevalence of potential off-target mutations caused by the CRISPR/Cas9 reagents and to allow recovery from the genetic bottleneck associated with single founder events. After back-crossing, each strain was self-crossed to obtained homozygous individuals, with genotypes determined again by HRMA and Sanger-based sequencing. Whereas homozygous SGS1^Δ13^/ SGS1^Δ13^ mosquitoes were readily obtained, SGS1^Δ25^ homozygotes did not appear to be viable (likely due to linkage to an unknown recessive lethal), since only heterozygotes and wild type genotypes were recovered from that cross despite repeated attempts. SGS1^Δ13^ homozygotes were crossed with SGS1^Δ25^ heterozygotes, followed by selection and self-crossing to the transheterozygous progeny. The resulting strain, containing a mixture of SGS1^Δ13^/ SGS1^Δ25^ transheterozygotes and SGS1^Δ13^/ SGS1^Δ13^ homozygotes was then referred to as SGS1^ko^, and was used for all subsequent experiments (proteomic analysis and parasite challenge).

### mRNA expression analyses

Real-time quantitative PCR (RT-qPCR) was performed using whole-body total RNA isolated from *A. aegypti* with TRIzol (Thermo Fisher Scientific, Waltham, USA), treated with DNAse I (Thermo Fisher Scientific, Waltham, USA) and quantified in a NanoDrop (Thermo Fisher Scientific, Waltham, USA) spectrophotometer. Generation of cDNA and subsequent amplification of each target transcript was performed using the OneStep RT-PCR kit (QIAGEN, Hilden, Germany) with 2 μg of treated RNA and primers listed in Additional file 1: Table S1, with the reaction mixture incubated at 50 °C for 30 min and 95 °C for 15 min. Amplification conditions were 94 °C for 1 min followed by 30 cycles of 94 °C for 1 min, 60 °C for 1 min and 72 °C for 1 min and a final step of 10 min at 72 °C. RT-qPCR was carried out using the isolated RNA following treatment with ezDNAse (Thermo Fisher Scientific, Waltham, USA) and quantification using a SpectraMax (Molecular Devices, Sunnyvale, CA, USA) spectrophotometer. One microgram of DNase-treated RNA was used to synthesize cDNA using SuperScript IV VILO Master Mix (Thermo Fisher Scientific, Waltham, USA) following the manufacturer’s protocol. RT-qPCR was performed with SsoAdvance Universal SYBR Green Supermix (BioRad, Hercules, USA) on a CFX96 Touch Real-Time PCR Detection System (BioRad, Hercules, USA). Reactions were performed in technical triplicates and two biological replicates, with 1/50 diluted cDNA, following the cycling parameters: 30 s at 95 °C, 45 cycles of 15 s 95 °C, 15 s at 60 °C and 10 s at 72 °C, and melting curve analyses at 65–95 °C. The dCT method was used to calculate expression relative to the rpS7 gene [32]. All primers used were designed on Primer 3 server (version 0.4.0) [33, 34] generating amplicons that ranged between 100-130 bp and an amplification efficiency verified to be 0.9–1.0. The primers are listed in Additional file 1: Table S1.

### Proteomic analyses

For both SGS1^KO^ and LVP control mosquitoes, three biological replicates, each consisting of five pairs of salivary glands dissected from sugar fed 5–7-day-old adult females in Phosphate-Buffered Saline (PBS) pH 7.4, were placed in a tube and frozen at −80 °C until use. Samples were submitted to liquid chromatography coupled with mass spectrometry (LC–MS) at the Research and Technology Branch (NIAID, NIH). Proteomic analyses were performed as described previously [35]. The number of unique mapping peptides was tabulated for each sample and the resulting matrix entered into EdgeR [36] for differential expression analysis. Only proteins with 6 or more unique peptides in at least three of the six samples were retained in the analysis. P-values were adjusted for multiple testing, with a final false discovery rate (FDR) set at 0.01.

### Quantification of Plasmodium infection/invasion of the mosquito

To analyse the prevalence and intensity of *Plasmodium* infection, adult females that had fed on parasite-infected blood had their midguts dissected at 6 days post-feeding. Midguts were stained with 0.1% (wt/vol) mercurochrome in water and oocysts counted by light microscopy. To quantify sporozoites in mosquito haemolymph, females had legs removed from one side and 1–5 μL of PBS was intrathoracically injected from the other side and a first drop (0.4–1 μL) of diluted haemolymph was recovered from the holes where the legs were, with a sterile pipette. Haemolymph was pooled from mosquitoes until 10 μL was recovered then placed in a haemocytometer and sporozoite numbers determined by phase-contrast microscopy. The collection was done 8 days after an infected blood meal. For sporozoite quantification in mosquito salivary glands*,* salivary gland pairs were dissected 8 days after a *P. gallinaceum*-infected blood meal. Dissected salivary glands were transferred to a drop of PBS and carefully washed, collected and placed in tubes containing 10 μL of fresh PBS, and were individually homogenized by pipetting. Sporozoite numbers were determined in C-Chip^™^ disposable haemocytometers (InCyto, Thermo Fisher Scientific, Waltham, USA) by phase-contrast microscopy. For CRISPR/Cas9 knockout challenge experiments, the second and the third replicates for both the salivary gland dissection and sporozoite counts were performed blinded. Essentially, the researchers aware of the genotypes did not perform the sporozoite counts, and the researchers dissecting and counting sporozoites did not know the genotypes. To do this, containers with LVP or SGS1^KO^ mosquitoes were colour-coded without genotype identifiers. Following dissection, a second blinding step occurred as tubes containing the dissected salivary glands were relabelled with randomized numbers and sporozoites counted. After the completion of the counts the genotypes were revealed and data analysed.

### Blood feeding and reproductive fitness

For probing time and duration of blood meal experiments SGS^ko^ or LVP mosquitoes were individually placed in a transparent vial covered with mesh. Identification labels containing the genotype were covered with opaque tape and randomized numbers assigned. The tubes were then placed in the order of the randomized number and provided to a separate investigator performing the experiments. Probing time was recorded by initiating the timer with the insertion of the proboscis into the skin of a volunteer (BBK or ZNA). The timer was stopped once blood was detected in the abdomen. The duration of blood meal was recorded by initiating the timer as soon as blood was detected in the abdomen and stopped once the female withdrew the proboscis from the skin. The recording was terminated if probing time reached 300 s. Following probing time and duration of blood meal assays, fecundity and fertility experiments were performed using the EAgaL plate method [37]. Briefly, blood fed females were kept in the vial for 3 days with raisins and then placed individually in agarose-coated wells of a 24-well plate and allowed to oviposit. Each well was photographed to allow counting of embryos and after 4 days the wells were flooded to allow hatching. Five days later the wells were photographed again and images analysed for larval counts. Only once the entire experiment was completed were genotypes revealed and the data analysed.

### Statistical analysis

D’Agostino-Pearson omnibus normality test was used to determine whether oocyst and sporozoite counts were normally distributed and either an unpaired t test or Mann–Whitney test (GraphPad Prism version 7.02 for Windows, GraphPad Software, La Jolla, USA, www.graphpad.com), were used accordingly to assess the statistical significance of the differences between control and experimental groups. A P value < 0.05 was considered statistically significant. The percent reduction in sporozoites or oocysts was calculated as 100 × {1 − [(mean number of parasites in the experimental group)/(mean number of parasites in the control group)]}.

## Results

### Knockdown of aaSGS1 lowers the number of sporozoites invading the salivary gland

**Fig. 1:**
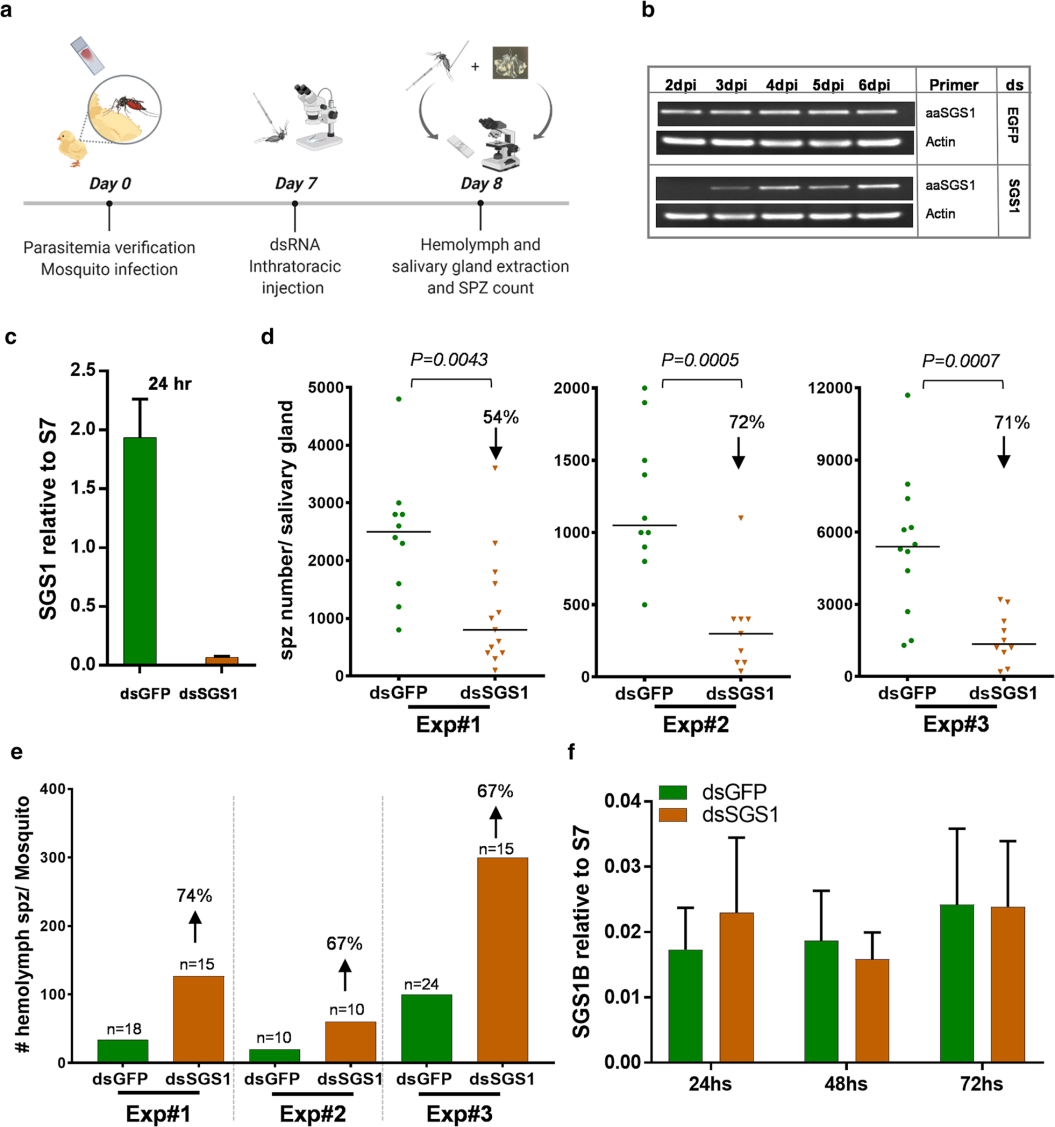
RNAi of *Aedes aegypti* SGS1 reduces the number of sporozoites invading the salivary glands. **a** Timeline of the knockdown experiment. **b** *aaSGS1* transcript detection from knockdown mosquitoes. Total RNA was extracted from pools of 5 females at 2, 3, 4, 5, and 6 days post injection (dpi) of dsSGS1 or dsEGFP mosquitoes and used in an RT-PCR reaction with specific primers for SGS1 or actin. **c** SGS1 normalized mRNA abundance by RT-qPCR from mosquitoes silenced by dsSGS1 or dsGFP at 24hs post injection; bars represent standard deviation from two replicates. **d** Salivary gland sporozoite intensity in dsRNA-treated, *P. gallinaceum-*infected *A. aegypti*. Each point represents one pair of salivary glands and the horizontal bars represents the median. A Mann–Whitney *U* test was used to evaluate statistical significance of parasite intensity of infection. A P value of  < 0.05 was considered statistically significant. **e** Mean haemolymph sporozoite intensity in dsRNA-treated, *P. gallinaceum-*infected *A. aegypti*. n represents the total number of females used to obtain haemolymph. **f** SGS1b normalized mRNA abundance by RT-qPCR from mosquitoes silenced by dsSGS1 or dsGFP; bars represent standard deviation from two technical replicates

Korochkina et al. [20] demonstrated that injection of a polyclonal antibody generated from *aaSGS1* could block sporozoite invasion of the salivary glands. As paralogs for this gene have since been described and were found to be expressed in the salivary glands, RNAi was used to determine the specific role of SGS1 in this process (Fig. 1a). To analyse the potential to silence *aaSGS1* as well as establish the duration of silencing, RNA was extracted from 5 females and transcript presence was determined in samples prepared 2, 3, 4, 5 and 6 days after intrathoracic injections of SGS1 dsRNA by RT-PCR and by RT-qPCR in samples prepared 24 and 48 h after injections. A decrease in *aaSGS1* transcript level was observed on day 2 post injection, however on day 3 the mRNA was detected, with expression essentially recovered on day 4 post injection (Fig. 1b). RT-qPCR confirmed silencing of SGS1 transcripts at 24 h post injection as well (Fig. 1c). These data suggest that the window of silencing obtained for this dsRNA is short, lasting for 48 h only. Therefore, dsRNA injections would have to be precisely timed to decrease the expression of SGS1 right when the first sporozoites were reaching the salivary gland, otherwise no effect would be detected. In order to confirm the specific role of SGS1 in the invasion of salivary glands by sporozoites, 3 µg of dsRNA was intrathoracically injected into *A. aegypti* seven days after a blood meal from a *P. gallinaceum* infected chicken, and on the next day salivary glands were dissected and haemolymph obtained with the corresponding number of sporozoites determined. Salivary gland-associated sporozoites were significantly reduced (67%) in mosquitoes injected with dsSGS1 in comparison to the controls injected with dsEGFP (Fig. 1d). This impact on sporozoite invasion was only observed when 3 µg of dsRNA was injected; when 1.5 µg of dsRNA was used, no statistically significant difference in the sporozoite numbers between dsSGS1 and controls could be detected although a reduction was observed (Additional file 1: Fig. S1). A potential increased number (though not statistically significant), of sporozoites was observed in the haemolymph of SGS1 knockdown mosquitoes, consistent with a potential inability to invade the salivary glands and therefore accumulation in the haemolymph (Fig. 1e).

### aaSGS1 gene silencing is specific and does not affect its orthologue aaSGS1b

A phylogenetic tree of SGS genes showed that SGS1 has several paralogs within the *A. aegypti* genome. The closest is a gene termed *aaSGS1b* (AAEL009992—historical identifier/AAEL029057—current identifier), which is located adjacent to SGS1 in the *A. aegypti* genome and is likely the result of a tandem duplication. While overall identity between SGS1 and SGS1b is only 46%, these proteins share large stretches of highly conserved residues, particularly at the membrane-bound C-terminal region [22]. To exclude the possibility that the dsRNA targeting SGS1 might also interfere with SGS1b, RT-qPCR was performed to evaluate the level of expression of SGS1b in female mosquitoes injected with dsRNA targeting SGS1 or EGFP. No difference in the expression of SGS1b could be detected in mosquitoes injected with dsSGS1 or dsEGFP (Fig. 1f), confirming that dsSGS1 is specific to silence *aaSGS1* and does not interfere with *aaSGS1b* expression.

### Generation of SGS1 knockout lines

**Fig. 2:**
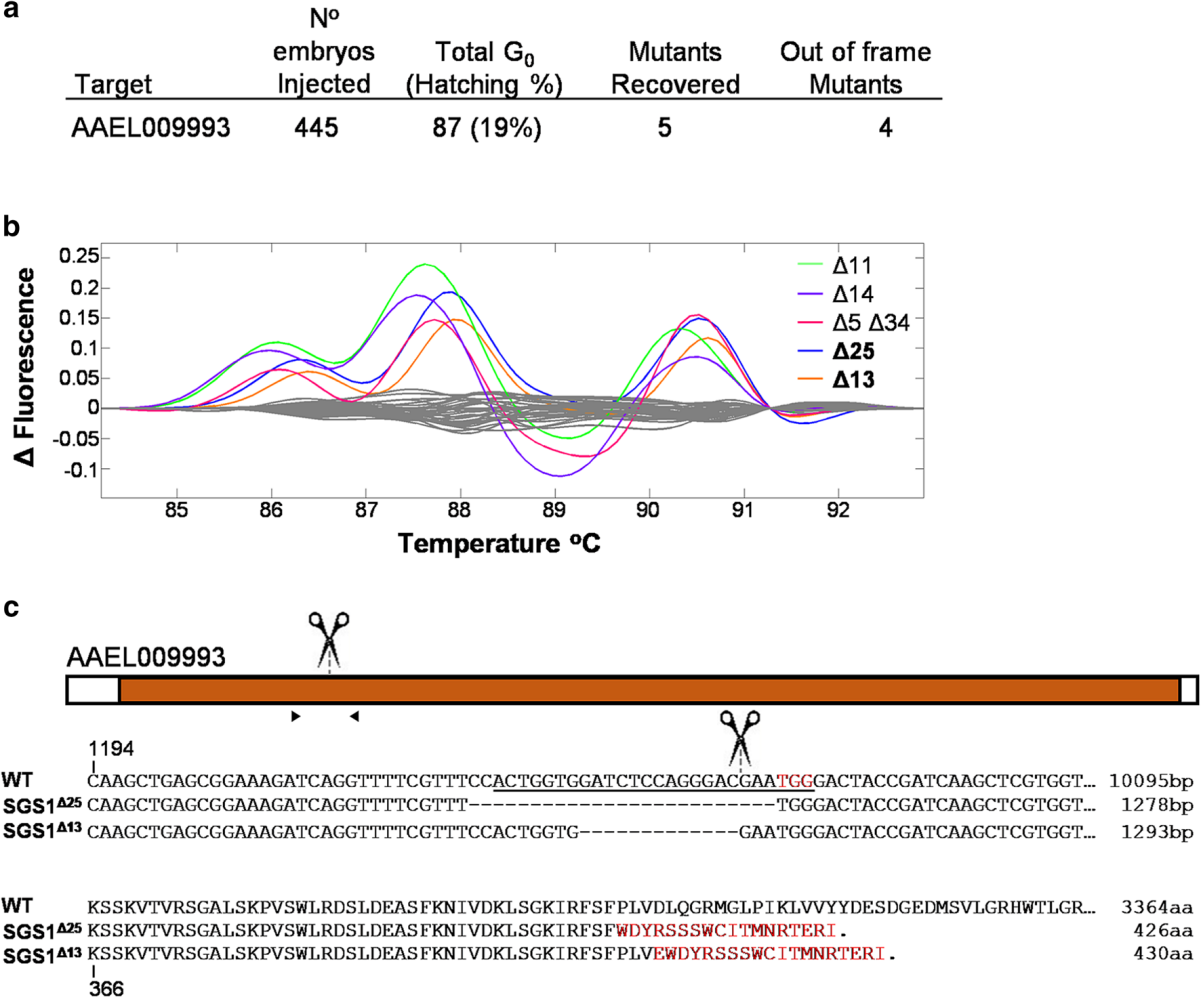
Generation of SGS1 knockout lines. **a** Number of embryos injected and mutant individuals obtained. **b** HRMA curve analyses of DNA extracted from legs of G_1_ male mosquitos. Coloured lines represent mosquitoes with indels confirmed by sequencing. Gray lines represent wild-type LVP mosquitoes. **c** Gene structure of *aaSGS1* along with nucleotide and protein alignment of the knockout lines generated by CRISPR/Cas9. Arrows represent the primer set used for genotyping in HRMA. Wild-type (WT) LVP and mutant SGS^Δ25^ and SGS^Δ13^ sequences are indicated. The sgRNA target sequence is underlined; scissors icon indicates the cleavage point with the PAM marked in red. Deleted bases are represented by dashes on the alignment. The red residues are the predicted amino acids generated through the result in frameshift, with (.) indicating a termination codon

While RNAi-based depletion of SGS1 reduced the number of salivary gland sporozoites, it did not prevent invasion. This could be due to incomplete/temporary silencing of SGS1, or to the existence of SGS1-independent invasion processes. To differentiate between these possibilities, knockout lines using CRISPR/Cas9 were developed, in order to completely abolish SGS1 expression. Cas9 protein and three synthetic guide RNAs were complexed and injected into pre-blastoderm *A. aegypti* embryos (Fig. 2a). Surviving G_0_ adults were outcrossed to the parental strain and a subset of G_1_ progeny screened for indels using high resolution melt analysis (HRMA) (Fig. 2b). Five different mutations were recovered (Δ11, Δ14, Δ5 Δ34, Δ25, and Δ13) (Fig. 2b), four of which were predicted to disrupt the open reading frame of SGS1. Of these, two knockout lines, SGS1^Δ25^ and SGS1^Δ13^, were selected to proceed with for phenotypic characterization. Both mutations are predicted to result in the premature termination of the SGS1 protein, including all transmembrane domains, with just 430 and 426 amino acids expected for SGS1^Δ25^ and SGS1^Δ13^ respectively, instead of 3364 amino acids for the full SGS1 (Fig. 2c). After back crossing each line to LVP for 5 generations, both SGS1^Δ25^ and SGS1^Δ13^ were crossed as described in the materials and methods to generate the SGS1^ko^ strain.

**Fig. 3:**
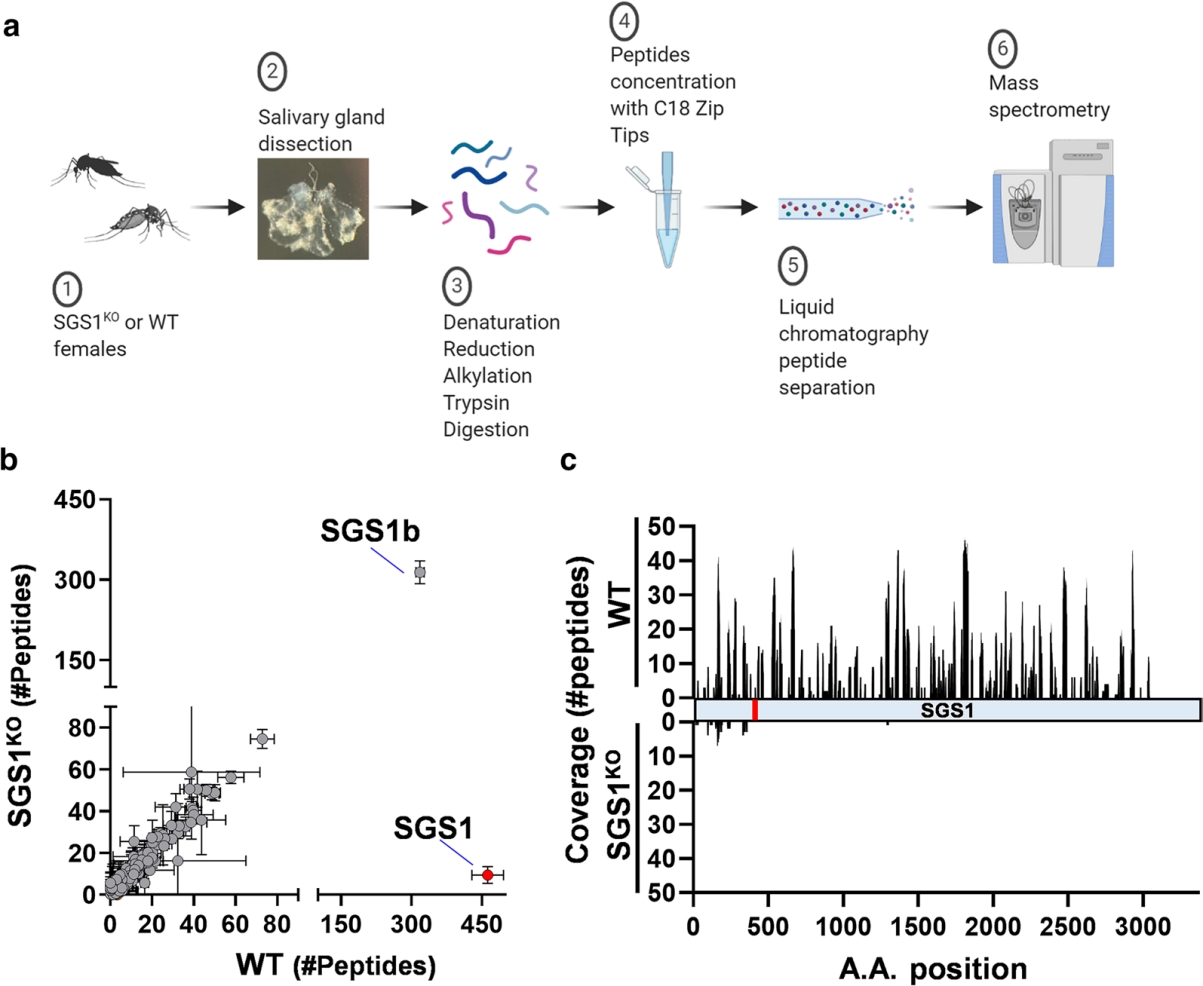
SGS1 protein expression is abrogated in SGS1^ko^ mosquitoes. **a** Diagram of workflow. **b** The number of unique-mapping peptides was linear normalized per 5000 peptides recovered and plotted for both WT and SGS1^ko^. Error bars represent the standard deviation based on three biological replicates for both WT (horizontal) and SGS1^ko^ (vertical). Proteins found to be differentially expressed through EdgeR analysis are shown in red. **c** Coverage of peptides recovered from proteomic analysis of salivary glands matching to SGS1 protein from wild-type (WT) and SGS1^ko^ mosquitoes. Red bar indicates the approximate position of the CRISPR/Cas9 target site

To verify that SGS1 was completely abolished in SGS1^ko^ mosquitoes, a proteomic analysis was performed using dissected salivary glands from SGS1^ko^ or LVP control females (Fig. 3a). More than 7000 uniquely mapping peptides from each of three replicate salivary gland samples were recovered (Additional file 2). In LVP salivary glands homogenates, about 10% of uniquely mapping peptides derived from SGS1 (peptide data presented are not normalized by size of the target protein, so a disproportionate number of peptides from SGS1 was expected due to its large size), while in SGS1^ko^ knockout mosquitoes the proportion was almost 100-times lower (Fig. 3b, Additional file 2). Differential expression analysis indicated no change in the proportion of peptides derived from any other salivary gland protein, including SGS1b, between salivary gland homogenates from SGS1^ko^ and LVP controls (Fig. 3b, Additional file 2). As some peptides mapping to SGS1 were still recovered from SGS1^ko^ mosquitoes, it is possible that these might derive from the N-terminal portion of the protein upstream of the CRISPR-induced frameshift. Indeed, while peptides obtained from the LVP strain mapped over almost the entire length of SGS1, those from SGS1^ko^ mosquitoes clustered at the N-terminus (Fig. 3c). Together, this evidence suggests that SGS1^ko^ mosquitoes are deficient in SGS1 protein.

**Fig. 4:**
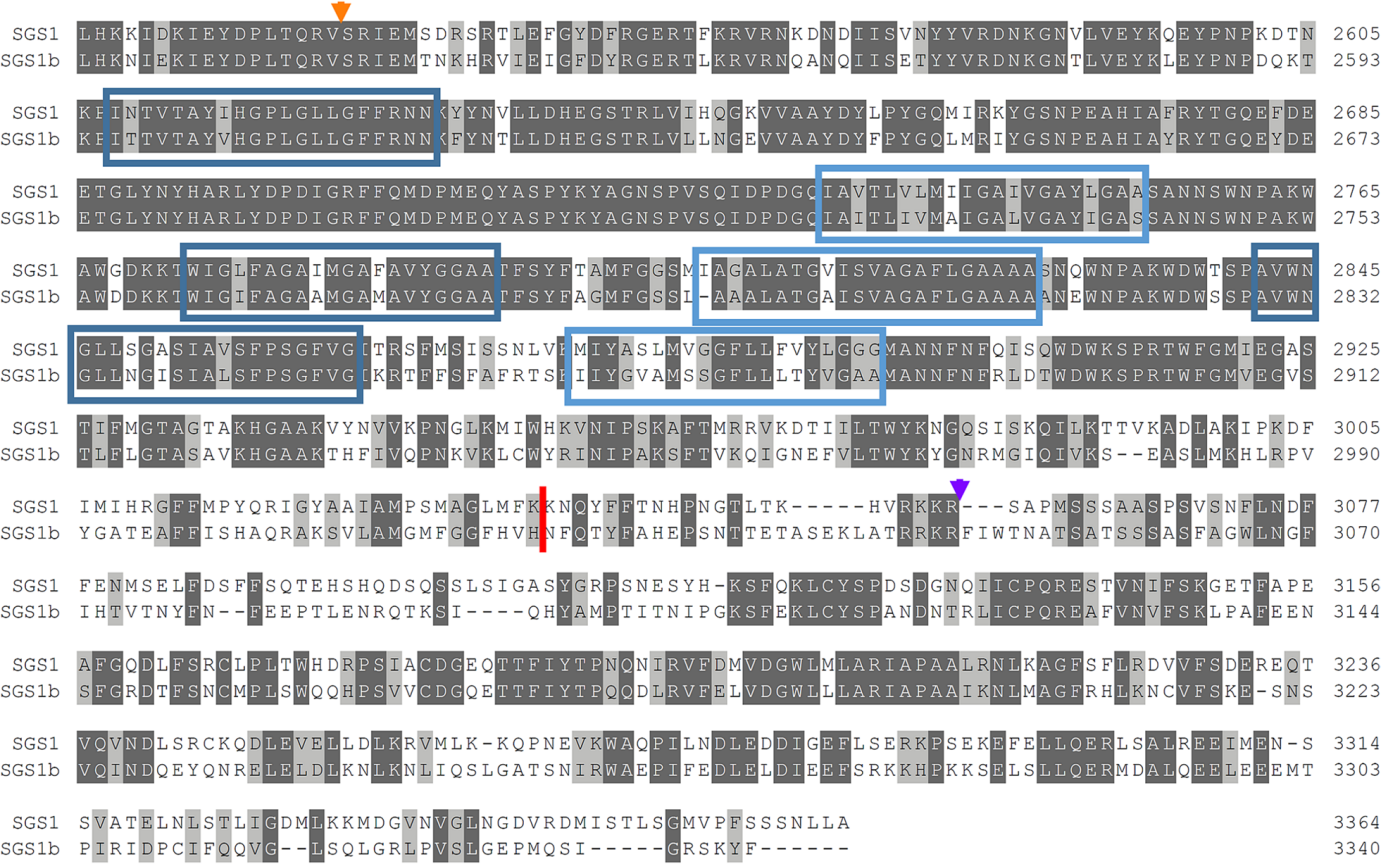
Alignment of *Aedes aegypti* SGS1 and SGS1b C-terminal regions. Amino acid sequences were aligned using MUSCLE as implemented in MEGA7 [40]. Alignment was imported into Multiple Align Show (https://www.bioinformatics.org/sms/multi_align.html). Dark/Light blue boxes indicate predicted transmembrane domains [41]. Red bar indicates the boundary after which no peptides were recovered from salivary glands in WT *A. aegypti* samples. Arrowheads indicate potential PAP (orange) or furin (purple) cleavage sites predicted in both SGS1 and SGS1b [42]

**Fig. 5:**
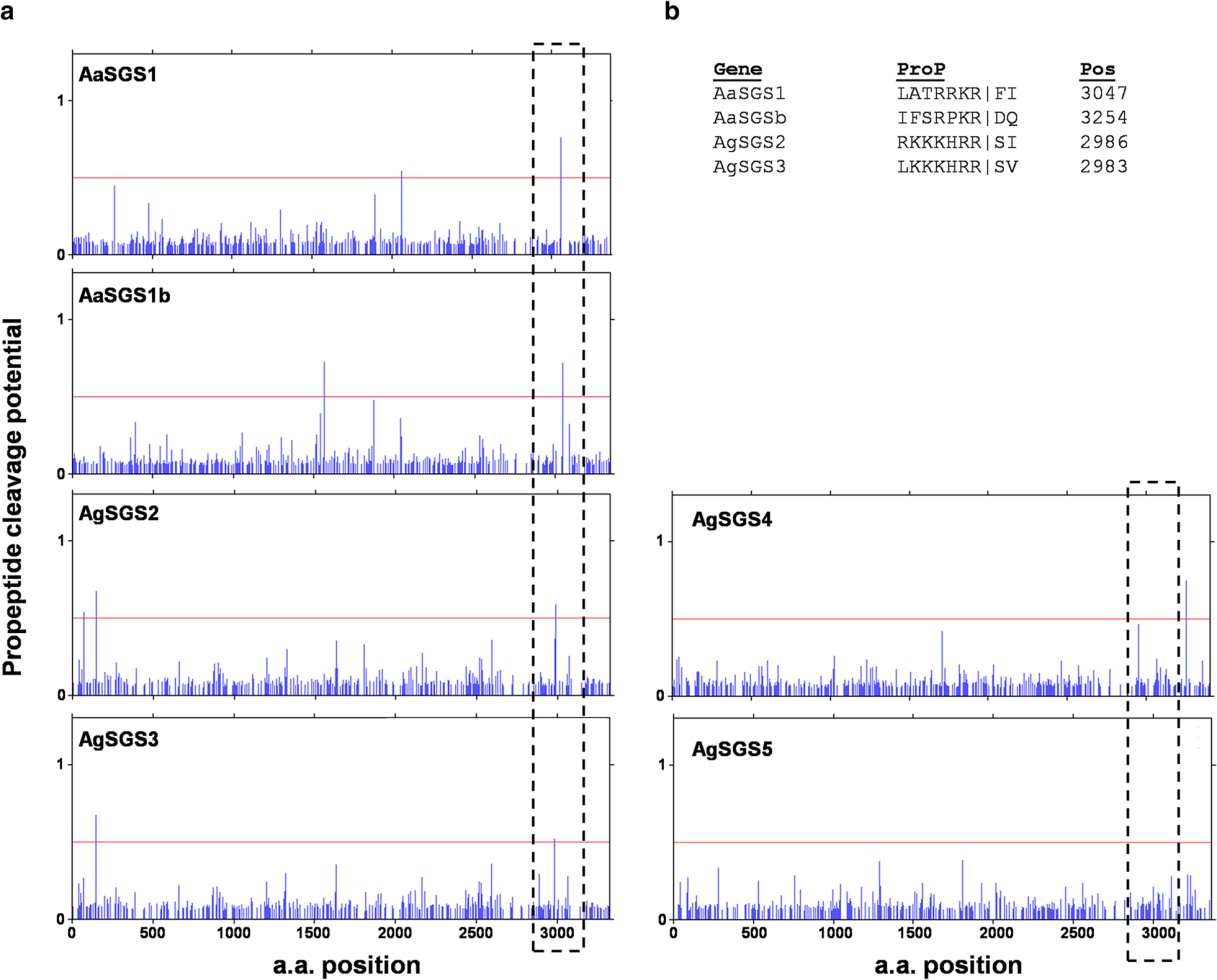
Prediction of furin-type cleavage sites in SGS proteins. **a** Probability of furin-mediated cleavage at each amino acid position in SGS proteins from *A. aegypti* (AaSGS1, AaSGS1b) and *An. gambiae* (AgSGS2, AgSGS3, AgSGS4, AgSGS5). Red horizontal line indicates cleavage is more likely than not; dotted box region highlights conserved prediction at the C-terminus. **b** Predicted furin cleavage site and position in SGS proteins

Interestingly, in wild-type LVP mosquitoes, no peptides were mapped to the C-terminus of SGS1 after position 3036 (Fig. 3c, Additional file 2). As the ORF is predicted to continue for another 329 residues, and this region remains conserved in SGS1b (Fig. 4), a search was performed for potential post-translational proteolytic cleavage sites that could explain the lack of peptides from this region. Two furin cleavage sites were predicted across the length of the SGS1, only one of which was conserved in SGS1b (Figs. 4, 5). This site sits just 22 residues after the last detected peptide (Fig. 4), and it also appears to be conserved in *Anopheles gambiae AgSGS2* and *AgSGS3*, but not in the salivary-gland specific *AgSGS4* and *AgSGS5* (Fig. 5). Together, these results suggest that SGS1 may be post-translationally processed into three polypeptides, only two of which remain associated with the salivary glands.

### Fitness of *SGS1*^*ko*^ mosquitoes

**Fig. 6:**
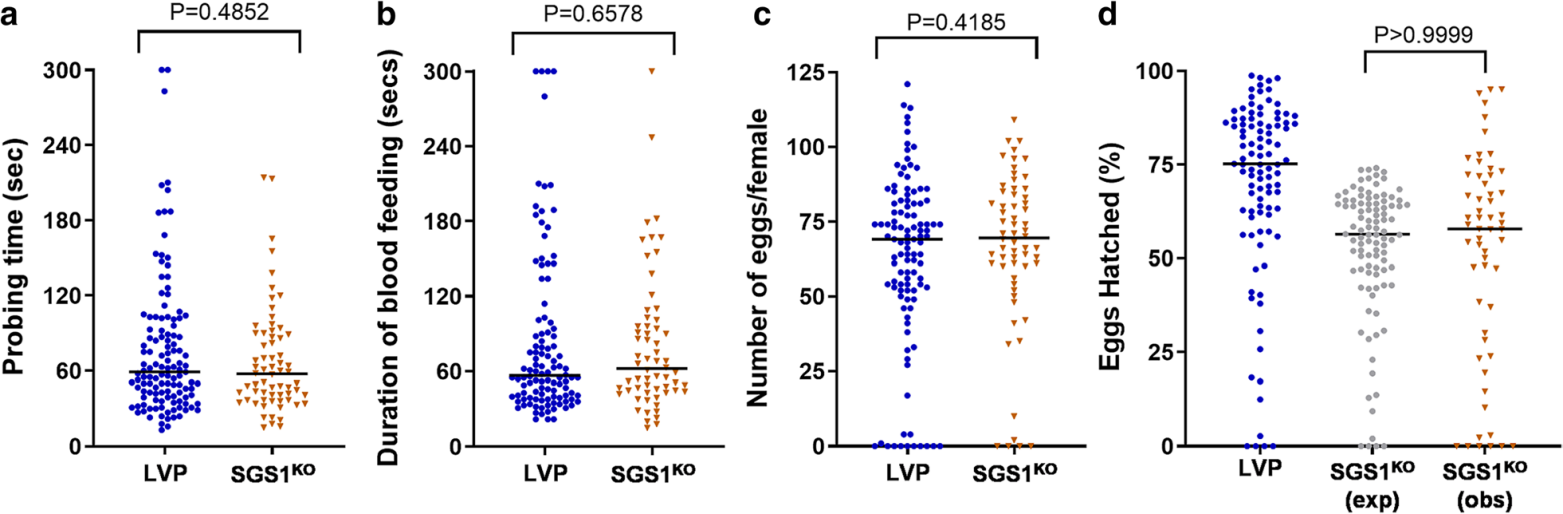
Effect of SGS1 knockout on bloodfeeding and female reproductive traits. **a** Probing time, **b** Duration of a blood meal, **c** Fecundity and, **d** Fertility for individual *SGS*^*ko*^ and LVP control mosquitoes. For **a–c**, the Mann–Whitney test was used to assess statistical significance between control and experimental groups; a P value of  < 0.05 was considered statisticaly significant. For **d** each value for the LVP group was converted into an expected value based on the assumption of a reduction in hatch rates of 25% due to the inviability of SGS1^Δ25^ homozygotes. All three groups were compared with a one way ANOVA (P < 0.00001), with the Kruskal–Wallis test applied to find differences between groups. P-value indicates the comparison between SGS1^ko^ expected (exp) and observed (obs) groups. Horizontal bars represent the median value; each experiment was performed four times, with the combined aggregated data shown

As SGS1 is expressed specifically in the female salivary glands, alterations in the blood feeding behavior of SGS1^ko^ mosquitoes were investigated. No significant difference was observed between SGS1^ko^ and LVP in terms of probing time or duration of blood feeding (Fig. 6a-b). As salivary gland proteins can be ingested and potentially impact blood digestion or nutrient absorption [38], both the fecundity and fertility of SGS1^ko^ mosquitoes were also evaluated. Both SGS1^ko^ and LVP females produced the same number of eggs (Fig. 6c). As expected, a reduced hatch rate was observed from SGS1^ko^ in comparison to LVP controls, since it was already established that SGS1^Δ25^ homozygotes were non-viable. As these genotypes were expected to be present in 25% of SGS1^ko^ progeny, the hatch rates of LVP controls were adjusted to simulate a 25% reduction in hatch for each mosquito. As shown in Fig. 6d, the observed hatch rate for SGS1^ko^ matched the expected hatch rate almost exactly, indicating that loss of SGS1 had no effect on female fertility. Overall, these data suggest that within the parameters investigated, loss of SGS1 does not represent a significant deleterious fitness effect to female *A. aegypti.*

### Knockout of SGS1 in *Aedes aegypti* impacts *Plasmodium gallinaceum* development

**Fig. 7:**
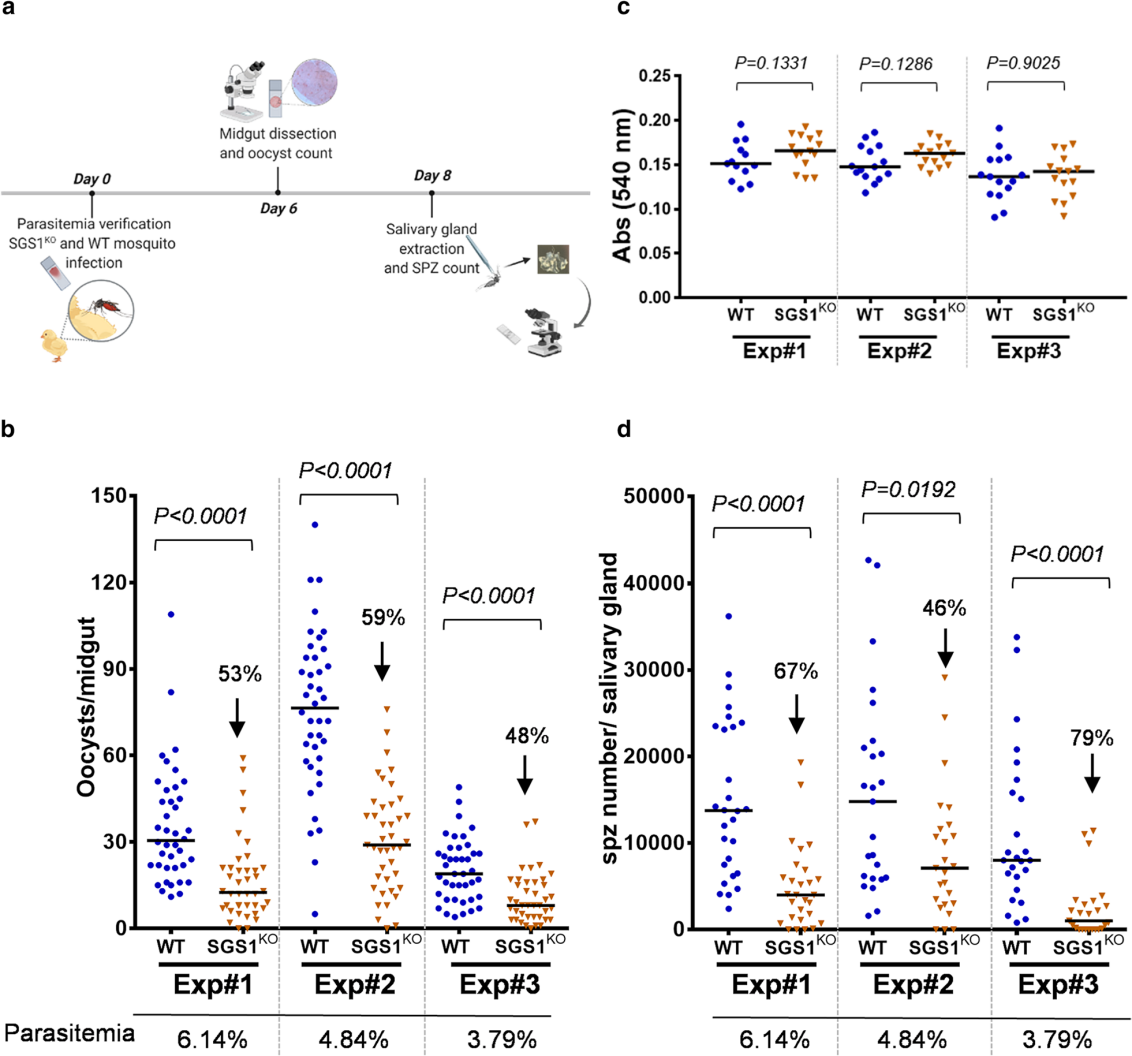
*Aedes aegypti* SGS1 has a dual role into *P. gallinaceum* life cycle in the mosquito. **a** Timeline of *P. gallinaceum* challenge experiment in SGS1 knockout mosquitoes. **b** Oocyst intensity in the midgut of SGS1^ko^ or LVP infected mosquitoes, each point represents one midgut and the horizontal bar represents the median. **c** Hemoglobin concentration in midguts of *P. gallinaceum*-challenged mosquitoes. Each point represents the absorbance (Abs) from a single homogenized midgut from either wild-type (WT) or SGS1 knockout (SGS1^KO^) mosquitoes. **d** Sporozoite prevalence in the salivary gland of SGS1^ko^ or LVP infected mosquitoes. Each point represents one pair of salivary glands. Horizontal bars represent the median. A Mann–Whitney *U* test was used to evaluate statistical significance of parasite intensity of infection. A P value of  < 0.05 was considered statisticaly significant

The effect of SGS1 knockout on parasite infection and invasion of the mosquito salivary glands was next determined. SGS1 knockout and LVP control mosquitoes were allowed to feed on *P. gallinaceum* infected chicks. After 6 days, a subset of mosquitoes were dissected and scored for the number of midgut oocysts, while the remaining were dissected at day 8 and scored for salivary gland sporozoites (Fig. 7a). Unexpectedly, a significant reduction (53%) in oocyst number was found in SGS1^ko^ mosquito midguts in comparison to the LVP control in all three independent experiments (Fig. 7b) from chickens with different levels of parasitaemia (3.79–6.14%). The lower number of parasites could be due SGS1^ko^ mosquitoes imbibing less blood from the infected chickens as compared to wild-type, and so the hemoglobin content in the midguts of blood fed females was quantified. No difference was observed between LVP and SGS1^KO^ females (Fig. 7c), indicating that both groups were exposed to similar parasite numbers. Thus, the decrease in oocyst numbers in SGS1^ko^ mosquitoes suggests a novel role for SGS1 in parasite development during the oocyst stage. Loss of SGS1 was also associated with an average of 64% reduction in salivary gland sporozoites in all three independent experiments as compared to LVP control (Fig. 7d). Notably, for the third replicate in which the chicken used to infect mosquitoes had the lowest parasitaemia and parasite prevalence, the oocyst reduction found in SGS1^ko^ mosquitoes was about the same as the others experiments (approximately half compared to the LVP controls, Fig. 7b), however the number of sporozoites were drastically reduced, with a higher percentage of salivary glands with zero sporozoites not found in any LVP control mosquitoes (28% in SGS1^ko^ on the third replicate *versus* 10% and 12% in SGS1^ko^ on the first and second replicate respectively) (Fig. 7d). In contrast, during the second replicate in which parasite prevalence was the highest of the three experiments, oocyst reduction was also approximately half in comparison to the LVP controls (Fig. 7b). However, the decrease in sporozoite numbers was the lowest observed, not reaching half of sporozoite numbers from SGS1 knockouts mosquitoes in relation to LVP controls (Fig. 7d). Together, this may indicate that while SGS1 has a role in facilitating invasion of *P. gallinaceum* into the mosquito salivary glands, this can be overwhelmed by higher infection intensities. In summary, SGS1 has a dual role in the *P. gallinaceum* life cycle, one in oocyst development and another in facilitating salivary gland invasion in *A. aegypti*.

## Discussion

This report describes the knockdown and the knockout of SGS1 in the mosquito *A. aegypti* and its role in the *P. gallinaceum* life cycle. Silencing SGS1 with the use of double stranded RNA could only be achieved transiently with large amounts of dsRNA injected into mosquitoes. That was not surprising, as other authors have described the same issue silencing mosquito salivary gland genes [39]. The duration of *SGS1* silencing was also short, lasting only 48hs, while *apyrase* and *SGL1L3* silencing lasted at least 13 days [39] indicating that the duration of the silencing effect is also gene specific. The specificity of silencing was evaluated in regards to *SGS1b*, a paralog of *SGS1*. No effect could be observed in *SGS1b* gene expression with the injection of dsSGS1, reiterating the specificity of dsSGS1 even with large amounts of dsRNA, a question raised by Boisson et al. [39] who observed the same specificity with different salivary gland genes also using large amounts.

The effective knockdown of *SGS1* indeed impacted the sporozoite penetration of the salivary gland, with 67% fewer sporozoites in the silenced salivary glands, confirming a role in facilitating salivary gland penetration. These findings corroborate results found by Korochkina et al*. *[20] in which polyclonal antibodies against SGS1 blocked about 65% of sporozoite invasion. Interestingly, polyclonal antibodies against the whole salivary gland extract displayed the same blocking effect as the antibody against SGS1 suggesting that the other molecules that may facilitate sporozoite invasion may not be protein based.

The next question addressed if completely abolishing *SGS1* expression could prevent sporozoite salivary gland penetration. CRISPR/Cas9 was used to successfully generate out of frame mutations in *aaSGS1*. SGS1 protein expression was lost in those mosquitoes as proteomic analyses by LC–MS of salivary glands indicated, however the impact on sporozoite penetration remained similar to knockdown experiments (67% in knockdown experiments, versus 64% in the knockout). This could indicate that the knockdown, although short-lived, was highly effective, potentially abrogating protein expression during the short window when the sporozoites were released from the oocyst until salivary gland invasion and the time of the dissections. It is unlikely that other factors compensate for the loss of SGS1 in knockout mosquitoes, as no other changes were observed in the overall expression of salivary gland proteins in the proteomic analyses. Therefore, the results presented here in addition to others [19] support the hypothesis that other factor/s present on the salivary gland surface also facilitate sporozoite penetration.

**Fig. 8:**
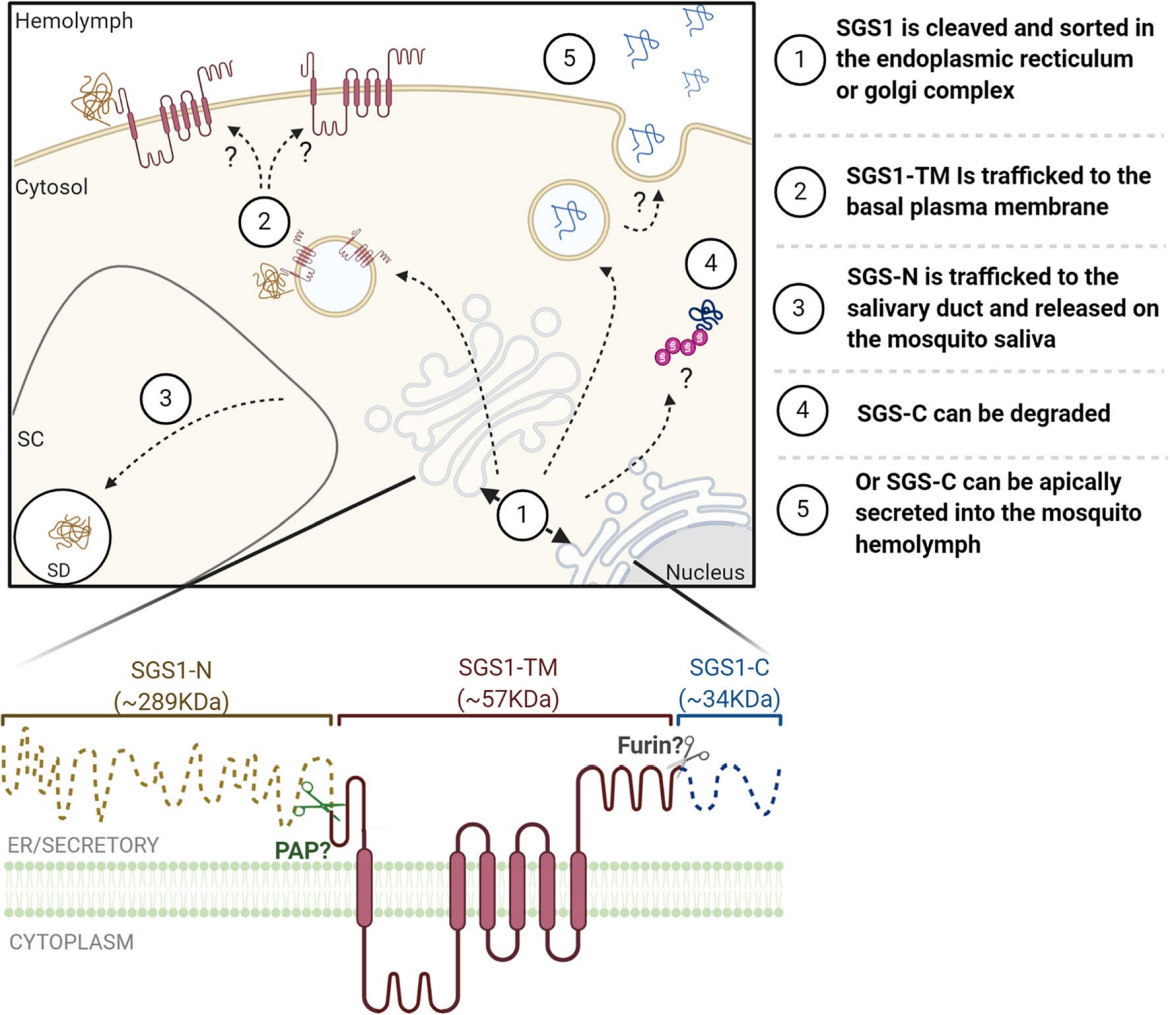
SGS1 proposed mechanism of cleavage and trafficking and schematic representation. Schematic representation of full lengh protein, putative peptides generated after cleavage and possible routes of peptide trafficking. The scissors represents the cleavage sites for PAP (prophenoloxidase-activating protease) and furin; SD, secretory duct; SC, secretory cavity

Surprisingly, SGS1 knockout mosquitoes also had reduced oocyst numbers (53%) in comparison to controls fed on the same infected chicks. These results were not anticipated, since SGS1 expression is limited to the salivary glands [20]. King et al. predicted the SGS1 protein to be post-translationally cleaved into a large N-terminal soluble fragment and a highly hydrophobic membrane-bound fragment containing at least six transmembrane helices based on the presence of a prophenoloxidase-activating protease (PAP) type cleavage site on the N-terminus of the transmembrane domain [22]. Western blot analysis against the N-terminus of the *An. gambiae* salivary-gland specific SGS4/5 or the N-terminal of the predicted transmembrane domain confirmed this post-translational cleavage, probably on the PAP site. However, the authors detected a much smaller C-terminal region than expected (~ 47 kDa rather than  ~ 85 kDa). While King et al. reasoned that the high hydrophobicity of that portion of the protein could cause excessive SDS-binding leading to a faster migration through SDS–polyacrylamide gels and appearance of less massive size band, another possible explanation is the presence of a second post-translational cleavage step. Indeed, a single potential furin cleavage site was identified in some SGS proteins following the C-terminal transmembrane domain, though interestingly not in *AgSGS4* or *AgSGS5*. It is possible that such sites are present, but failed to be predicted by the algorithm used. Taken together, this suggests that the *aaSGS1* gene actually encodes three separate peptides, referred to here as SGS1-N, SGS1-TM and SGS1-C (Fig. 8). SGS1-N is the large N-terminal fragment secreted into the salivary duct, passed into the saliva and present at the bite site during blood feeding [23], but if cleavage is incomplete may also be retained in the salivary gland basal plasma membrane [20]. SGS1-TM is trafficked to the basal plasma membrane of the salivary gland epithelium, and is potentially used by *P. gallinaceum* to facilitate sporozoite invasion. As no peptides corresponding to SGS1-C were recovered in whole salivary glands of wild type mosquitoes, it is possible that this fragment is secreted into the haemolymph. However, this remains hypothetical, as this fragment may be degraded or trafficked to another part of the mosquito body as well (Fig. 8). Thus, the unexpected effect of *aaSGS1* knockout on oocyst intensity may be mediated by secreted SGS1-N, which may interact with either parasite or the bite site, or both, during feeding, or by SGS1-C. A mass spectrometry based proteomic analysis of *A. aegypti* early midgut peritrophic matrix showed that 10% of the proteins identified corresponded to known salivary gland proteins [38], demonstrating that salivary proteins secreted at the bite site are re-ingested during blood feeding. This suggests another alternative where SGS1-N may mediate its effect on oocyst numbers at the midgut level.

## Conclusion

The data presented here support the role of SGS1 as participating in, but not essential for, invasion of *A. aegypti* salivary glands by *P. gallinaceum*, and also as facilitator of parasite development in the mosquito midgut. SGS1 could, therefore, be part of a strategy to decrease malaria transmission by the mosquito vector, for example in a transgenic mosquito that blocks its interaction with the parasite. SGS1-deficient mosquitoes had no measurable defects in blood feeding or female fertility, a somewhat surprising finding given the abundance of this protein in the saliva and its evolutionary conservation. It is possible that other salivary SGS proteins provide some level of functional redundancy, or that SGS1 proteins are of importance only in certain contexts that may be present in the wild but are not found in laboratory experiments. Future work will be necessary to understand the mechanism by which SGS1 enables oocyst development and salivary gland invasion by sporozoites, and additional mutant strains will help clarify the specific roles of SGS-N, SGS1-TM and SGS1-C in these processes.

## Supplementary Information

**Additional file 1: Table S1.** Oligonucleotides used in this study. **Fig. S1.** Thoracic injection of 1500 ng of dsSGS1 does not impact sporozoite penetration in salivary glands. Salivary gland sporozoite prevalence of *P. gallinaceum-*infected *A. aegypti* female silenced with 1500 ng of dsSGS1, each point represents one pair of salivary glands. A Mann–Whitney *U* test was used to evaluate statistical significance of parasite mean intensity of infection.

**Additional file 2:** Proteomic analyses of salivary glands extract of LVP and SGS1^KO^ mosquitos

## Data Availability

All data generated or analysed during this study are included in this published article [and its supplementary information files].
