# Supplementary material for: Aedes aegypti SGS1 is critical for Plasmodium gallinaceum infection of both the mosquito midgut and salivary glands
Source: Malar J. 2021 Jan 6;20:11. doi: 10.1186/s12936-020-03537-6 (PMC7787129; doi:10.1186/s12936-020-03537-6)
Supplement: Supplementary file 1 — Additional file 1: Table S1. Oligonucleotides used in this study. Fig. S1. Thoracic injection of 1500 ng of dsSGS1 does not impact sporozoite penetration in salivary glands. Salivary gland sporozoite prevalence of P. gallinaceum-infected A. aegypti female silenced with 1500 ng of dsSGS1, each point represents one pair of salivary glands. A Mann–Whitney U test was used to evaluate statistical significance of parasite mean intensity of infection. [file 12936_2020_3537_MOESM1_ESM.pptx]

## Slide 1
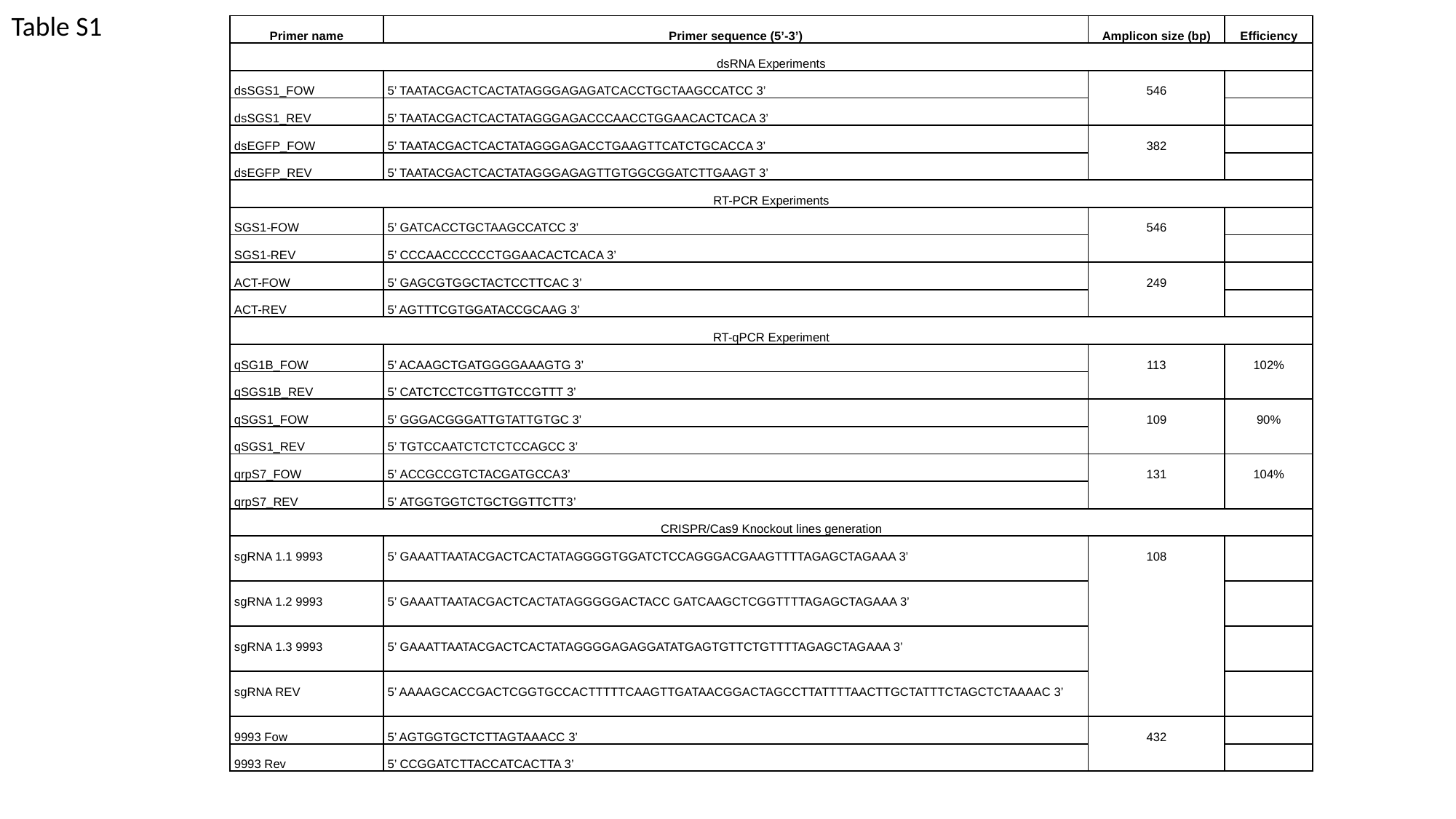

Table S1
| Primer name | Primer sequence (5’-3’) | Amplicon size (bp) | Efficiency |
| --- | --- | --- | --- |
| dsRNA Experiments | | | |
| dsSGS1\_FOW | 5’ TAATACGACTCACTATAGGGAGAGATCACCTGCTAAGCCATCC 3’ | 546 | |
| dsSGS1\_REV | 5’ TAATACGACTCACTATAGGGAGACCCAACCTGGAACACTCACA 3’ | | |
| dsEGFP\_FOW | 5’ TAATACGACTCACTATAGGGAGACCTGAAGTTCATCTGCACCA 3’ | 382 | |
| dsEGFP\_REV | 5’ TAATACGACTCACTATAGGGAGAGTTGTGGCGGATCTTGAAGT 3’ | | |
| RT-PCR Experiments | | | |
| SGS1-FOW | 5’ GATCACCTGCTAAGCCATCC 3’ | 546 | |
| SGS1-REV | 5’ CCCAACCCCCCTGGAACACTCACA 3’ | | |
| ACT-FOW | 5’ GAGCGTGGCTACTCCTTCAC 3’ | 249 | |
| ACT-REV | 5’ AGTTTCGTGGATACCGCAAG 3’ | | |
| RT-qPCR Experiment | | | |
| qSG1B\_FOW | 5’ ACAAGCTGATGGGGAAAGTG 3’ | 113 | 102% |
| qSGS1B\_REV | 5’ CATCTCCTCGTTGTCCGTTT 3’ | | |
| qSGS1\_FOW | 5’ GGGACGGGATTGTATTGTGC 3’ | 109 | 90% |
| qSGS1\_REV | 5’ TGTCCAATCTCTCTCCAGCC 3’ | | |
| qrpS7\_FOW | 5’ accgccgtctacgatgcca3’ | 131 | 104% |
| qrpS7\_REV | 5’ atggtggtctgctggttctt3’ | | |
| CRISPR/Cas9 Knockout lines generation | | | |
| sgRNA 1.1 9993 | 5’ GAAATTAATACGACTCACTATAGGGGTGGATCTCCAGGGACGAAGTTTTAGAGCTAGAAA 3’ | 108 | |
| sgRNA 1.2 9993 | 5’ GAAATTAATACGACTCACTATAGGGGGACTACC GATCAAGCTCGGTTTTAGAGCTAGAAA 3’ | | |
| sgRNA 1.3 9993 | 5’ GAAATTAATACGACTCACTATAGGGGAGAGGATATGAGTGTTCTGTTTTAGAGCTAGAAA 3’ | | |
| sgRNA REV | 5’ AAAAGCACCGACTCGGTGCCACTTTTTCAAGTTGATAACGGACTAGCCTTATTTTAACTTGCTATTTCTAGCTCTAAAAC 3’ | | |
| 9993 Fow | 5’ AGTGGTGCTCTTAGTAAACC 3’ | 432 | |
| 9993 Rev | 5’ CCGGATCTTACCATCACTTA 3’ | | |

## Slide 2
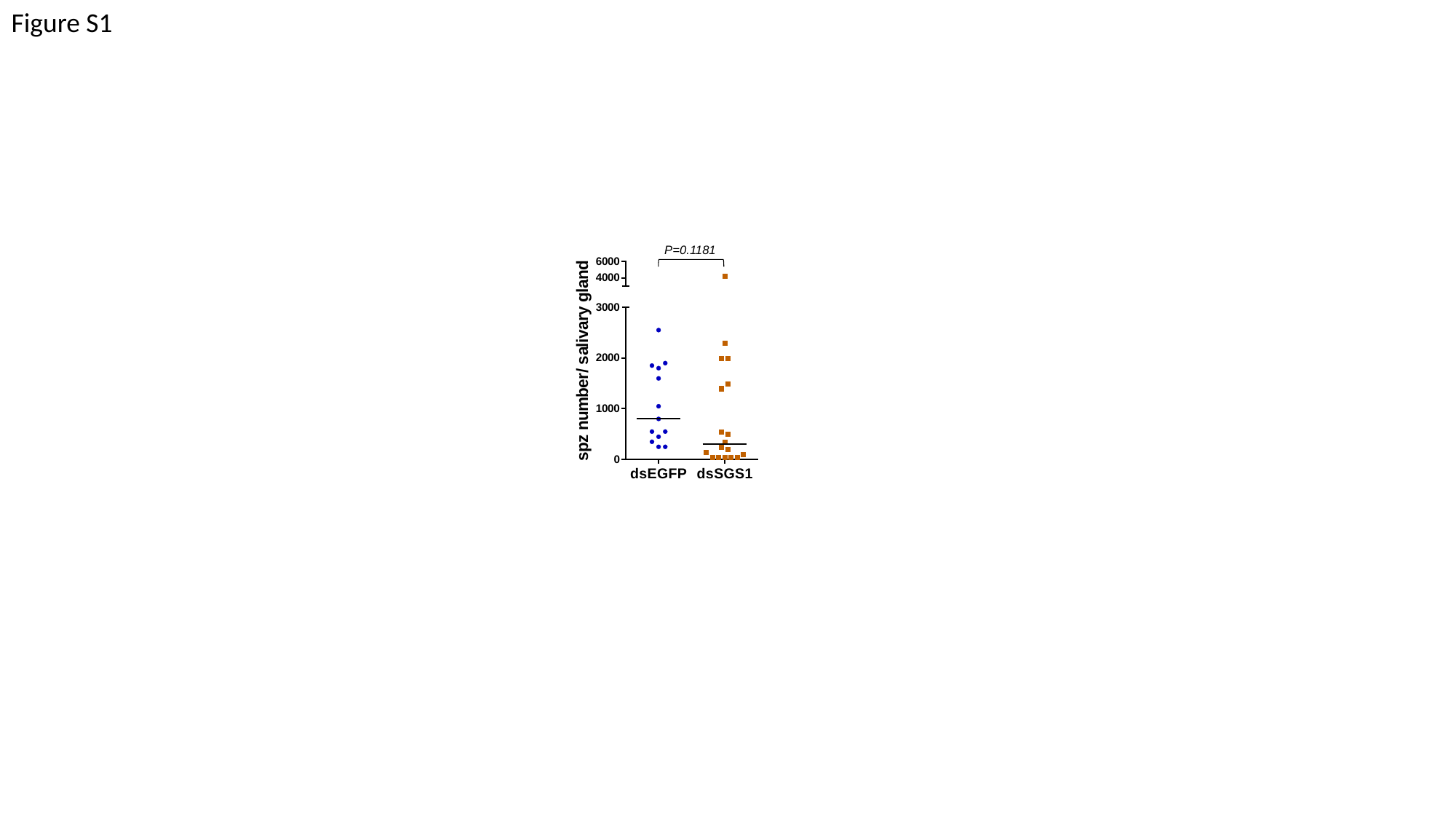

Figure S1
P=0.1181
